# Negative feedback of SNRK to circ-SNRK regulates cardiac function post-myocardial infarction

**DOI:** 10.1038/s41418-021-00885-x

**Published:** 2021-10-07

**Authors:** Zhi-Yan Wang, Xiao-Xiao Liu, Yun-Fei Deng

**Affiliations:** 1 Department of cardiovascular medicine, Ruijin Hospital, Shanghai Jiao Tong University School of Medicine, Shanghai, China; 2 Department of Cardiology, Shanghai Institute of Cardiovascular Diseases, Zhongshan Hospital, Shanghai Medical College of Fudan University, Shanghai, China; 3 Department of Cardiology, Nanjing First Hospital, Nanjing Medical University, Nanjing, China

**Keywords:** Cell biology, Gene regulation

## Abstract

A limited delivery of oxygen and metabolic substrate to the heart caused by myocardial infarction (MI) impairs the cardiac function, and often results in heart failure. Here, we identified a circRNA (circ-SNRK) from SNRK (sucrose nonfermenting 1-related kinase, which can increase the cardiac mitochondrial efficiency) in cardiomyocytes (CMs). Circ-SNRK can sponge the miR-33 and in turn improved the ATP synthesis via SNRK, proving the existence of circ-SNRK - miR-33 - SNRK axis. Furthermore, we found that protein NOVA1 (NOVA alternative splicing regulator 1) could accelerate the circ-SNRK formation; a cleaved peptide (~55 kDa) from SNRK enters the nucleus and blocks the cyclization of circ-SNRK via binding to NOVA1. The aforementioned negative feedback of SNRK to circ-SNRK limited the SNRK at a proper level, and inhibited the protective role of circ-SNRK in ischemic heart. In addition, our in vivo experiment indicated that the overexpression of exogenic circ-SNRK could break this loop and improves the cardiac function post-MI in rats. Together, our results demonstrated that the negative loop of circ-SNRK with SNRK regulates the energy metabolism in CMs, thus might be a potential therapeutic target for heart failure.

## Introduction

Heart failure (HF) is a chronic and progressive condition and a major healthcare problem worldwide. It is a frequent complication of myocardial infarction (MI) caused by acute or persistent occlusion of the coronary artery. Given that the adult heart exhibits a limited regeneration ability, it is believed that studies on the prevention of cardiomyocytes (CMs) loss post-MI might decrease the incidence of HF.

As a new member of non-coding RNA, circular RNA (circRNA) is mainly produced by back-splicing from exons with a covalent bond. The trait of resisting exonuclease digestion makes circRNA much more stable than linear RNA [1]. CircRNA mainly residues in cytoplasm and exerts function acting as a miRNAs sponge [2]. Nevertheless, some circRNA, namely ciRNAs (circular intron RNAs) or EIciRNAs (exon-intron RNAs) can be found in nucleus and affect the transcription of their parental genes serving as the cis-acting elements [3, 4]. In addition, some studies indicated that the circRNA is intensively associated with cardiovascular diseases via acting as a miRNAs sponge [5–7]. Yet, to date, few studies focused on the relationship between circRNA and HF post-MI.

SNRK (Sucrose nonfermenting related kinase) is a member of AMP-activated protein kinase (AMPK) family. Unlike other members, it can be activated by LKB1 (liver kinase B1) without additional subunits or stimuli [8]. Previous studies showed that SNRK is associated with energy metabolism and cardiac function [9–11]. Also, a more recent study suggested that SNRK is regulated by miR-103a-3p and in turn affects the renal inflammation and fibrosis [12]. Here, we discovered that circ-SNRK (derived from SNRK) significantly decreases in hypoxia-treated CMs (in vitro) or post-MI heart (in vivo). It could improve the ATP synthesis by increasing SNRK protein via miR-33. In vivo results indicated that overexpressed circ-SNRK could ameliorate the deterioration of cardiac function post-MI, implying that it might a promising therapeutic target for HF post-MI.

## Results

### The identification of circ-SNRK

**Fig. 1:**
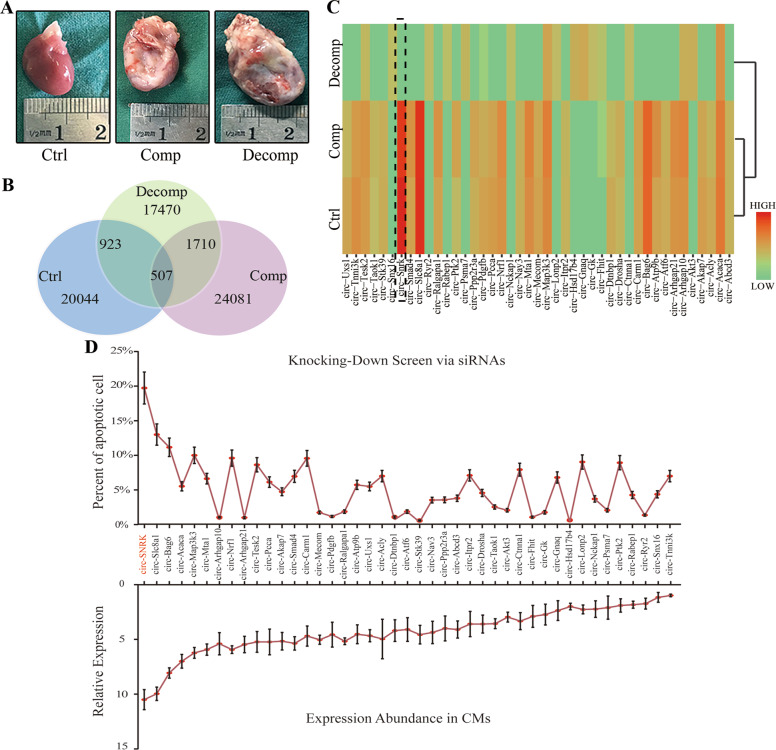
The identification of circ-SNRK. **A** Representative images of hearts from three groups at four or six weeks after surgery. **B** Venn diagram showing 507 circRNAs, which coexist between Ctrl group *VS* Decomp group (923) and Comp group *VS* Decomp group (1710). **C** Clustered heatmap showing the filtered differentially expressed 43 circRNAs in three groups. Black dotted box indicates the circ-SNRK. **D** Line chart showing the effects of 43 circRNAs on cell apoptosis (Upper) being downregulated by siRNAs and their relative expression abundance (Lower) in CMs (the lowest expression was normalized as 1). Data were expressed as mean ± SD, *n* = *3*.

The ligation of left anterior descending coronary artery (LAD) was utilized to establish rat HF model, evidenced by echocardiography and the general shape of hearts (Fig. 1A/Supplementary Fig. S1A). Based on the *echocardiographic* measurements, rats were classified into 3 groups (3 rats/group, details in methods): control (Ctrl), compensatory (Comp) and decompensatory (Decomp) group [13, 14]. The hearts were collected postoperatively at 4 (Ctrl and Comp group) or 6 weeks (Decomp group). Next, same amount of total RNA extracted from the heart in each group was mixed into CTRL-MIX, COMP-MIX and DECOMP-MIX, respectively to reduce the individual variation; then they were high-throughput sequenced to examine the circRNA expression.

Overall, 20044, 24081 and 17470 circRNAs were found in Ctrl, Comp and Decomp group, respectively according to algorithm CIRCexplorer [15]. Considering that Ctrl and Comp rats have the similar cardiac function, we filtered out 923 or 1710 differentially expressed (aFC (fold change) >2.0 and *p* < 0.05) circRNAs between groups (Ctrl *vs* Decomp group and Comp *vs* Decomp group, respectively). Moreover, 507 circRNAs coexisted in 923 (55%) and 1710 (30%) circRNAs; 98% (498) of 507 circRNAs showed similar expression abundance in Ctrl and Comp group (Fig. 1B). Given that some circRNAs are associated with the transcription of their parental gene and it also shares the pre-mRNA (precursor mRNA) with linear RNA, we concluded that circRNA is to some extent related to the function of their linear counterparts and then employed the GO and KEGG analysis for their parental genes to narrow the candidate circRNAs (Supplementary Fig. S1B) [3, 4, 16]. Results showed that 43 circRNAs were involved in ischemia-related pathways (including ATP binding and glucagon signal) (Fig. 1C, Supplementary Table S1). Finally, the FACS assay for cell apoptosis (the most common way of CMs loss post-MI) was used to screen these 43 circRNAs (downregulated by siRNAs). Particularly interesting was circ-SNRK that shows relatively high expression in CMs and is intensively related to cell apoptosis (Fig. 1D).

### The characteristics of circ-SNRK and its effects on CMs

**Fig. 2:**
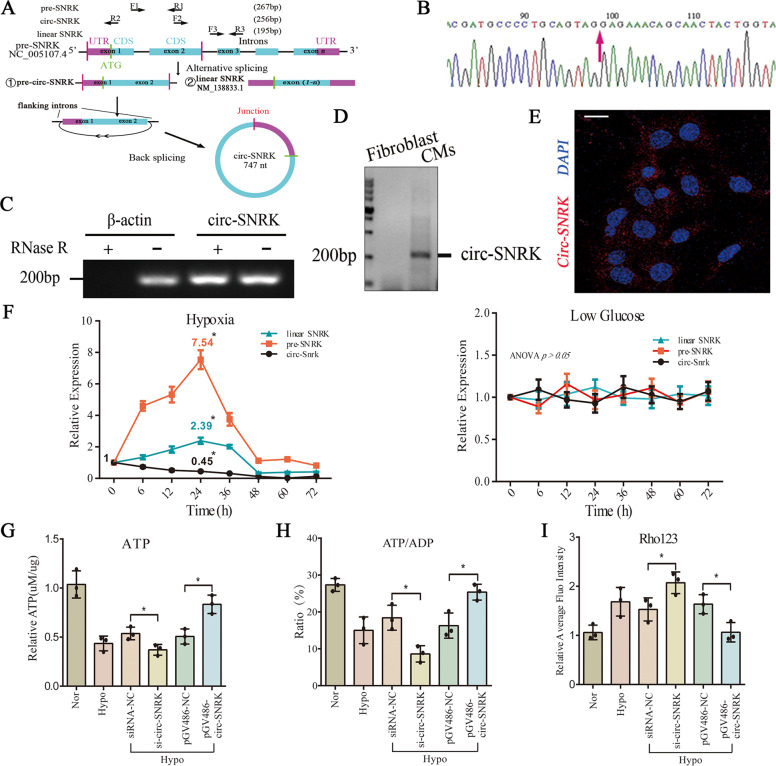
The traits of circ-SNRK and its effects on CMs. **A** Schematic representation of circ-SNRK generated from SNRK gene. The divergent primer sets (F2/R2) was used to detect circ-SNRK, and the convergent primer sets (F1/R2, F3/R3) were used to detect pre-SNRK (precursor SNRK) (F1/R1) and linear SNRK (mRNA) ((F3/R3 – F1/R1) because F3/R3 can detect both pre-SNRK and linear SNRK). Sanger sequencing (**B**) of PCR (F2/R2) products from NRCMs, and agarose gel electrophoresis analysis (**C**) of PCR products of circ-SNRK and β-actin from NRCMs with RNase R digestion or not. Red arrow indicates the junction. **D** Agarose gel electrophoresis showed PCR (F2/R2) products from CMs and fibroblast. **E** FISH assay of circ-SNRK in CMs with probes targeting the junction of circ-SNRK labeled with PE. Scale bar = 20 um. **F** Levels of circ-SNRK, linear SNRK and pre-SNRK in hypoxia (Left) or low glucose (Right)-treated NRCMs at different time points. Data were expressed as mean ± SD, *n* = 3, one-way ANOVA, *p* > 0.05, *p < 0.05. The ATP production, ATP/ADP ratio (**G**, **H**) and relative MMP (mitochondrial membrane potential) (**I**) of NRCMs with *pGV486-circ-SNRK or si-circ-SNRK*. Rho 123 reflects the anti-trend of MMP. Data were expressed as mean ± SD, *n* = 3, two-tailed *t*-test, *p < 0.05.

Circ-SNRK (747 nt) derived from the exon 1-2 of SNRK decreased significantly in the HF hearts based on RNA-seq data. Its existence in CMs was verified by Sanger sequencing and RNase R digestion (Fig. 2A–C). In addition, the successful amplification (F4/R4) of ~750 bp products and slower migration speed of circ-SNRK relative to its linear counterpart further supported that (Supplementary Fig. S2A, B). Our results also indicated that circ-SNRK expresses in diverse organs, residues in cytoplasm and does not change with age (Fig. 2D, E/ Supplementary Fig. S2C–E). Moreover, its expression abundance is ~3% to linear SNRK and > 0.1% to β-actin; and it is highly conserved among multiple species (Supplementary Fig. S2F). Meanwhile, We discovered an analog of circ-SNRK (hsa_circ_0004089 in *circBase (*http://www.circbase.org/*)*, 95% similar to circ-SNRK) in human heart. Taken together, its enrichment in CMs and high conservation suggested that circ-SNRK might play a vital role in heart.

qRT-PCR showed that circ-SNRK remarkably decreases in failing rat hearts, consistent with the RNA-seq data (Supplementary Fig. S2G). The main influence of heart ischemia is the insufficiency of oxygen and glucose to CMs. Our data showed that linear SNRK (mRNA) and pre-SNRK (precursor mRNA) in hypoxia-treated (94% N_2_, 5% CO_2_ and 1% O_2_) NRCMs (neonatal rat cardiomyocytes) increase and then decrease, while circ-SNRK shows only a decreasing trend. However, low glucose (<1 g/L) had no obvious impacts on them (Fig. 2A/F).

To address its physiological function, we up- or downregulated the circ-SNRK with *pGV486* or siRNA, respectively. Northern blot and RT-PCR (F4/R4) indicated that circ-SNRK could be detected in *pGV486-circ-SNRK* transfected HEK293 cell (not contain circ-SNRK originally); and only circ-SNRK (not pre-SNRK or linear SNRK) was downregulated by *si-circ-SNRK* (Supplementary Fig. S3A–D). Its formation ratio (relative to linear SNRK) reached the peak (~25%) at ~24 h after transfection. Its degradation ratio was lower than linear SNRK (Supplementary Fig. S3E, F). Consequently, we found that circ-SNRK could increase the energy production (Fig. 2G–I/Supplementary Fig. S4A–B), ameliorate the cell apoptosis, but has no obvious effects on cell autophagy and pyroptosis (Supplementary Fig. S4C–E). Following research clarified that circ-SNRK inhibits cell apoptosis acting as the energy regulator via SNRK protein [11], leading us mainly focus on its physiological function of energy regulator (Supplementary Fig. S4F).

### MiR-33 bridges circ-SNRK with ATP synthesis via SNRK

Given that circ-SNRK residues in cytoplasm, it likely exerted its function via the polypeptides translated from it or acting as a miRNAs sponge. After finding it contains an ORF (open reading frame), we cloned the reconstructed circ-SNRK with His tag into *pGV486*. And linearized ORF plus His tag acted as a positive control(Supplementary Fig. S5A). Immunoblot and immuno-staining showed that no peptide (~32 kDa) is observed, suggesting that circ-SNRK cannot be translated into a peptide in CMs (Supplementary Fig. S5B, C).

**Fig. 3:**
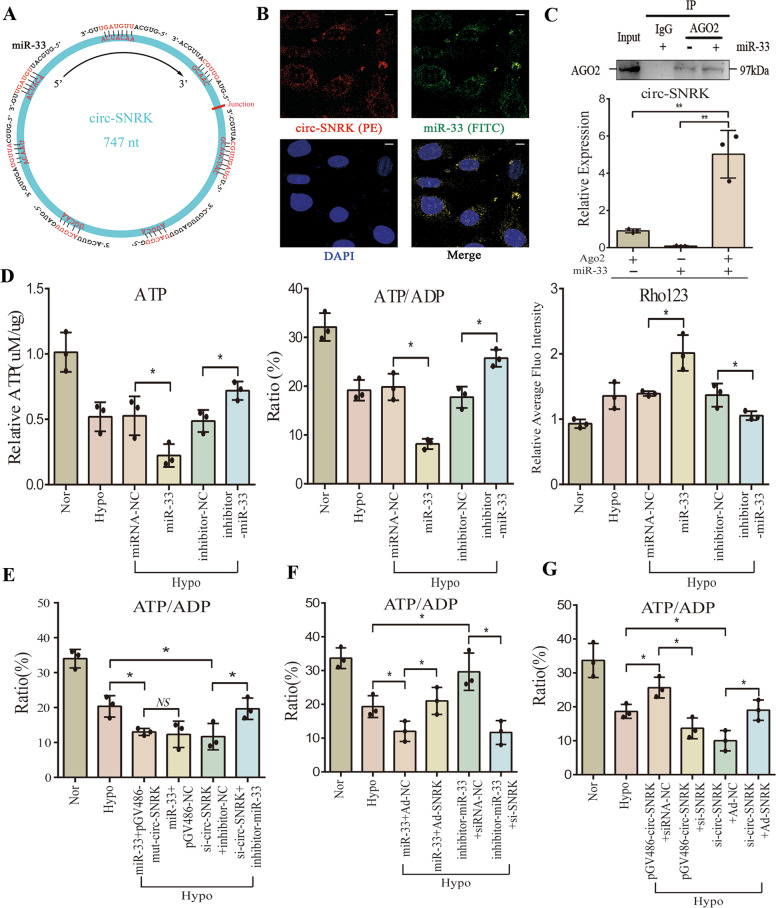
MiR-33 bridges circ-SNRK with ATP synthesis via SNRK. **A** Schematic of the 7 potentially binding sites of miR-33 in circ-SNRK. **B** FISH assay of circ-SNRK (red) and miR-33 (green) in the cytoplasm compared to the nucleus (blue). The probe of circ-SNRK was labeled with PE and the probe for miR-33 was labeled with FITC. Scale bar = 10 um. **C** (Upper) IP (Immunoprecipitation) of AGO2 in NRCMs with or without miR-33 overexpression; (Lower) qRT-PCR analysis of pulled-down circ-SNRK with AGO2 in NRCMs transfected with miR-33 or not. **D** The ATP production (Left, Mid) and MMP (Right) of NRCMs transfected with miR-33 mimic or inhibitor. **E** The ATP/ADP ratio of NRCMs co-transfected with *pGV486-mut-circ-SNRK* and miR-33 mimic or *si-circ-SNRK* and miR-33 inhibitor. **F** The ATP/ADP ratio of NRCMs co-transfected with miR-33 and *Ad-SNRK* or miR-33 inhibitor and *si-SNRK*. **G** The ATP/ADP ratio of NRCMs co-transfected with *pGV486-circ-SNRK* and *si-SNRK* or *si-circ-SNRK* and *Ad-SNRK*. **C**–**G** All data were expressed as mean ± SD, *n* = 3, two-tailed *t*-test, *p < 0.05, **p < 0.01.

Then, we discovered that miR-33 might be an ideal candidate for the presence of seven possible binding sites in circ-SNRK (Fig. 3A). Dual-luciferase reporter gene and FISH assays confirmed that circ-SNRK directly binds to miR-33 (Fig. 3B/Supplementary Fig. S6A, B). Meanwhile, we performed a RIP of AGO2 and found that the pulled-down circ-SNRK is much higher compared to the control group, but lower than that in miR-33 overexpressed CMs, further supporting their direct connection (Fig. 3C).

Considering that miRNA could degrade its target via RISC (RNA-induced silencing complex), we explored whether circ-SNRK or linear SNRK are degraded by miR-33. qRT-PCR showed a significant increase in circ-SNRK, while no obvious changes are observed for linear SNRK (Supplementary Fig. S6C), thus implying that miR-33 does not degrade them.

Next, we found that miR-33 could decrease the ATP synthesis and MMP in CMs, while its inhibitor has an opposite effect (Fig. 3D). Furthermore, the effects of *mut-circ-SNRK* (no miR-33 binding sites) on ATP synthesis and MMP were similar to *pGV486-NC*, whereas the effects of circ-SNRK reduction were rescued by miR-33 inhibitor (Fig. 3E/Supplementary Fig. S6D). These results indicated that miR-33 mediates the impacts of circ-SNRK on CMs. To further address the underlying mechanism, we predicted the downstream target of miR-33 via bioinformatics analysis and found that 3’UTR of SNRK contains the binding sites of miR-33. Dual-luciferase reporter gene assay and immunoblot clarified that miR-33 could directly bind to SNRK 3’ UTR to suppress its translation (Supplementary Fig. S6E, F).

To explore whether miR-33 regulates the energy production via SNRK, we first tested the role of SNRK in CMs. Results showed that ATP production, MMP and cellular viability significantly increased in hypoxia-treated NRCMs transfected with *Ad-SNRK (adenovirus vector)*, whereas the reduction in SNRK has an opposite effect (Supplementary Fig. S7A–C). In addition, the related proteins of SNRK affecting ATP synthesis (Trib3 (Tribbles Homolog 3), PPARα (Peroxisome Proliferator-Activated Receptor alpha) and UCP3 were altered as the previous study (Supplementary Fig. S7D) [11]. To clarify the relationship between circ-SNRK and Trib3/PPARα/UCP3, we constructed a SNRK plasmid containing the mutated 3’ UTR (*pCMV-SNRK-mut-3’ UTR;* unrecognized by miR-33) to abrogate the effects of circ-SNRK on SNRK translation. Immunoblot results noted that the change in circ-SNRK has no obvious effects on Trib3/PPARα/UCP3, suggesting that it mainly affects Trib3/PPARα/UCP3 via protein SNRK (Supplementary Fig. S7E, F). We co-transfected hypoxia-treated NRCMs with miR-33 and *Ad-SNRK* or inhibitor and *si-SNRK*. Results showed that effects of miR-33 on ATP production are disrupted by SNRK overexpression, while its low-expression influences are inhibited by SNRK reduction (Fig. 3F/Supplementary Fig. S8A). Together, SNRK mediated the impacts of miR-33 on ATP synthesis.

Finally, we examined whether circ-SNRK regulates energy metabolism via SNRK. Briefly, we found that circ-SNRK overexpression could increase SNRK protein, and its low-expression decreases the SNRK in NRCMs. Meanwhile, the upregulation of SNRK caused by circ-SNRK was ameliorated by miR-33, while decreased SNRK was rescued by miR-33 inhibitor (Supplementary Fig. S8B, C). So, circ-SNRK affected the SNRK expression via miR-33. Then, we transfected hypoxia-treated NRCMs with *pGV486-circ-SNRK* and *si-SNRK* or *si-circ-SNRK* and *Ad-SNRK*. We discovered that the effects of circ-SNRK overexpression on ATP production are inhibited by SNRK knockdown, while the effects of its low-expression are recused by SNRK overexpression (Fig. 3G/Supplementary Fig. S8D). Furthermore, we observed no changes in miR-33 in hypoxia-treated NRCMs; however, the expression of miR-33 was significantly higher compared to circ-SNRK (miR-33: circ-SNRK = ~*5:1*; Supplementary Fig. S8E). The similar expression pattern (circ-SNRK sponges 7 miR-33 (miR-33: circ-SNRK = *7:1*)) inferred that they can greatly affect each other in CMs. Additionally, increased SNRK in hypoxia-treated CMs implied that protein SNRK is mainly dominated by linear SNRK (mRNA) (Supplementary Fig. S8F). To sum up, our data suggested that circ-SNRK can affect ATP synthesis via miR-33 - SNRK axis.

### NOVA1 promotes the circ-SNRK formation binding to flanking introns directly

**Fig. 4:**
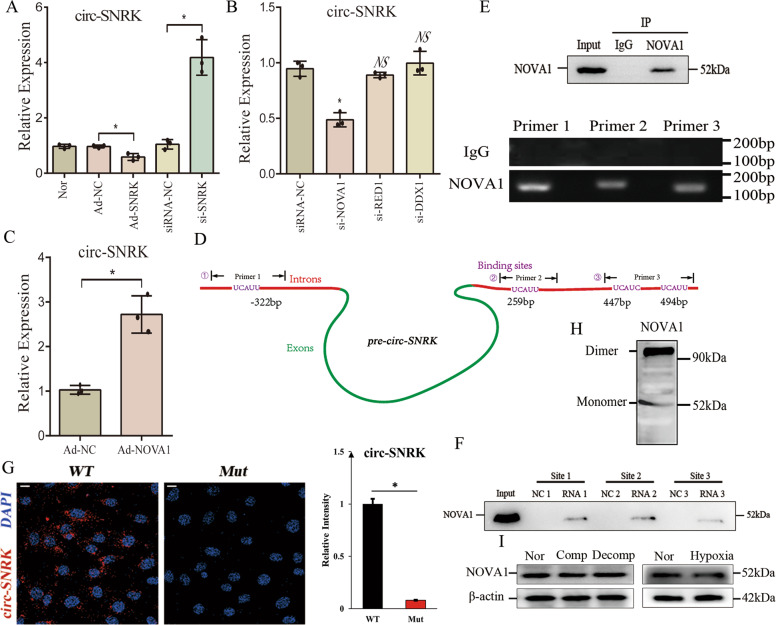
NOVA1 promotes the circ-SNRK formation binding to flanking introns directly. **A** qRT-PCR analysis of circ-SNRK in NRCMs with *Ad-SNRK* or *si-SNRK*. **B** qRT-PCR analysis of circ-SNRK in NRCMs transfected with *si-NOVA1, si-RED1* or *si-DDX1*. **C** The level of circ-SNRK in NRCMs with overexpressed NOVA1. **D** The schematic of pre-circ-SNRK. Primer 1, 2, 3 targeted the predicted four binding sites respectively, primer 3 covered 2 sites because they closed to each other. **E** RIP analysis of predicted 4 NOVA1-binding sites in introns of pre-circ-SNRK with NOVA1. (Upper) Co-immunoprecipitation (Co-IP) of endogenous NOVA1; (Lower) Agarose gel electrophoresis showed the PCR products (primer1, 2, 3) of RNAs pulled down by NOVA1. **F** Immunoblot analysis of proteins pulled down by biotin-labeled RNAs of predicted 4 NOVA1-binding sites with anti-NOVA1. **G** (Left) Confocal analysis of circ-SNRK (red) in HEK293 with upregulation of NOVA1 following be transfected with *pCMV-wt-pre-circ-SNRK* or *pCMV-mut-pre-circ-SNRK*. Scale bar = 10 um; (Right) Quantification of fluorescence intensity of circ-SNRK via ImageJ. **H** Native-PAGE analysis of total protein from NRCMs with anti-NOVA1. **I** Immunoblot of NOVA1 in hearts from different group or in NRCMs treated with hypoxia or not. **A**–**C**, **G** All data were expressed as mean ± SD, *n* = *3*, two-tailed *t*-test, *p < 0.05, NS not significant.

Previous reports showed that various factors could accelerate the formation of circRNA, including ALU elements, flanking complementary sequences, protein MBL (muscleblind) or protein quaking [1, 17–19]. By analyzing the sequences (500 nt) flanking circ-SNRK, we found that it has a low percentage of complementary sequences (~25%) or ALU elements (0%) and no binding sites of protein MBL or quaking. Given that miR-33 can increase the level of circ-SNRK and protein SNRK is the target of miR-33, we hypothesized whether SNRK affects the circ-SNRK cyclization to build a feedback (Supplementary Fig. S6C/E, F). qRT-PCR indicated that circ-SNRK is upregulated by *si-SNRK* and downregulated by *Ad-SNRK* in CMs (Fig. 4A), implying that SNRK plays a role in circ-SNRK formation.

Considering that SNRK has a reverse variation pattern with circ-SNRK, it should not directly connect to pre-circ-SNRK (precursor circ-SNRK, Fig. 2A) because the direct interaction will increase the circ-SNRK formation, like protein MBL or quaking. To identify the protein bridging SNRK with circ-SNRK, we analyzed the flanking introns (500 nt) of pre-circ-SNRK by *catRAPID* with a high score (*z-score* > *3*) to find the possible RNA-binding proteins (RBPs) [20]. 3 RBPs binding to both head and tail introns were filtered out, including NOVA1 (NOVA alternative splicing regulator 1), DDX1 (ATP-dependent RNA helicase DDX1) and RED1 (Double-stranded RNA-specific editase 1). SiRNAs targeting them were utilized to investigate which one could affect the circ-SNRK formation. qRT-PCR showed that only *si-NOVA1* could reduce the level of circ-SNRK (Fig. 4B). In addition, we detected an increase of circ-SNRK in CMs with overexpressed NOVA1 (Fig. 4C). Above results suggested that, unlike protein SNRK, NOVA1 could promote circ-SNRK formation.

NOVA1, an RNA-binding protein, could regulate the alternative splicing and bind specifically to the sequence UCAUY [21]. By analyzing the flanking introns of pre-circ-SNRK, we found 1 potential binding site of NOVA1 in 5’ intron and 3 in 3’ intron (Fig. 4D). To verify the interaction, RIP of NOVA1 was employed, and sequences from all predicted sites can be detected (Fig. 4E). Synthesized RNAs labeled with biotin acted as a bait to test whether NOVA1 can be pulled down, and all sites revealed positive results (Fig. 4F). Taken together, our data implied that NOVA1 can bind to the flanking introns of pre-circ-SNRK and affect the circ-SNRK formation.

To address whether NOVA1 is necessary to circ-SNRK formation, we cloned *pre-circ-SNRK* with NOVA1-binding sites (*wt* or *mut*) into plasmids (*pCMV-wt-pre-circ-SNRK, pCMV-mut-pre-circ-SNRK*), which were then transfected into *si-NOVA1* treated HEK293. Briefly, no circ-SNRK was observed in both conditions. However, the following overexpression of NOVA1 only rescued the circ-SNRK in *pCMV-wt-pre-circ-SNRK* transfected HEK293, thus suggesting the necessity of NOVA1 for circ-SNRK formation (Fig. 4G). Meanwhile, we discovered that it also could accelerate the formation of other circRNAs containing the NOVA1-binding sites, further proving the crucial role of NOVA1 in circRNA formation (Supplementary Fig. S9). Searching the NOVA1 structure in PDB (https://www.rcsb.org/), we found that NOVA1 has over two similar RNA-binding motifs. Also, the result of native PAGE showed that NOVA1 can form a dimer in NRCMs (Fig. 4H). Thus, we inferred that NOVA1 possesses the ability to promote the formation of circ-SNRK. Finally, we found that protein NOVA1 has no obvious variation in hypoxia-treated CMs and HF heart, suggesting that NOVA1 could exert its function in above conditions (Fig. 4I).

### A SNRK 55 kDa peptide connects NOVA1 to affect circ-SNRK formation

**Fig. 5:**
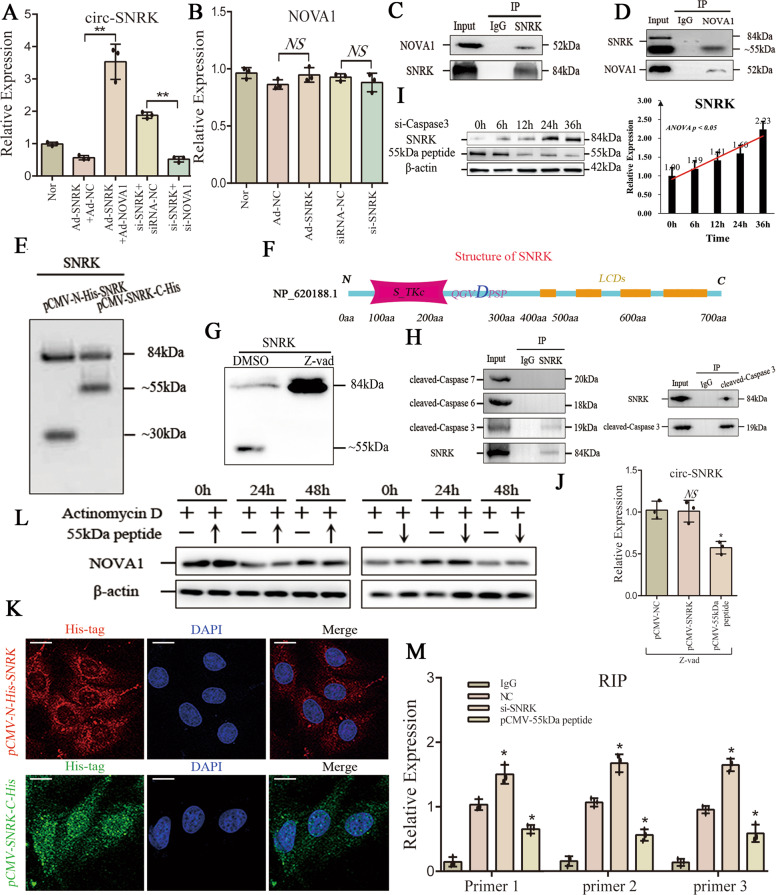
A 55 kDa peptide from SNRK connects with NOVA1 to affect circ-SNRK formation. **A** qRT-PCR analysis of circ-SNRK in NRCMs co-transfected with *Ad-SNRK* and *Ad-NOVA1* or *si-SNRK* and *si-NOVA1*. **B** The expression of NOVA1 mRNA in NRCMs with low- or overexpression of SNRK. **C** Co-IP of NOVA1 with SNRK in NRCMs. **D** Co-IP of SNRK with NOVA1 in NRCMs. **E** Immunoblot analysis of SNRK in NRCMs transfected with *pCMV-N-His-SNRK* or *pCMV-SNRK-C-His* using anti-His. **F** Schematic of SNRK structure. S_TKc Serine/Threonine protein kinases, LCDs low complex domains, D Aspartic acid. **G** Immunoblot of SNRK and its 55 kDa peptide in NRCMs treated with *Z-vad* or not. **H** (Left) Co-IP of activated Caspase 6/7/ 3 with SNRK in NRCMs; (Right) Co-IP of SNRK with activated Caspase 3. **I** (Left) The level of SNRK and 55 kDa peptide in NRCMs transfected with *si-Caspase 3* with time; (Right) Quantification of SNRK level normalized by β-actin via ImageJ. Data were expressed as mean ± SD, *n* = 3, *one-way ANOVA p* < 0.05. **J** The level of circ-SNRK in NRCMs with overexpression of SNRK or 55 kDa peptide. **K** Confocal analysis of anti-His in NRCMs transfected with *pCMV-N-His-SNRK* or *pCMV-SNRK-C-His*. Scale bar = 10 um. **L** Immunoblot of NOVA1 in Actinomycin D treated NRCMs with low- or overexpression of 55 kDa peptide with time. **M** RIP of introns flanking circ-SNRK (primer 1, 2, 3) in NRCMs transfected with *si-SNRK* or *pCMV-55kDa peptide*. Data were expressed as mean ± SD, *n* = 3, two-tailed *t*-test, *p < 0.05 *in relative to NC group*. **A**, **B**, **J** All data were expressed as mean ± SD, *n* = 3, two-tailed *t*-test, *p < 0.05, **p < 0.01, NS not significant.

Next, we examined whether NOVA1 acts as an intermediate between circ-SNRK and SNRK. qRT-PCR showed that NOVA1 overexpression could rescue the decrease of circ-SNRK caused by *Ad-SNRK*, whereas its low-expression had an opposite effect, suggesting that SNRK affects the circ-SNRK formation via NOVA1 (Fig. 5A/Supplementary Fig. S8G). To address the underlying mechanism, we first found that NOVA1 mRNA is not affected by SNRK protein (Fig. 5B). Then, we performed a co-IP of SNRK and discovered that NOVA1 is pulled down (Fig. 5C); but molecular weight of the pulled-down peptide by NOVA1 was ~55 kDa, which can be recognized by anti-SNRK, suggesting that it is a part of protein SNRK (Fig. 5D). Next, we constructed *pCMV-N-His-SNRK* and *pCMV-SNRK-C-His* to explore the peptide terminals and found two peptides: N-terminal peptide (~30 kDa) and C-terminal peptide (~55 kDa) evidenced by the liquid chromatography tandem MS (mass spectrum) (Fig. 5E/Supplementary Fig. S8H). Analyzing the structure of SNRK via *SMART* (http://smart.embl-heidelberg.de/), we discovered that protein SNRK is made of a catalytic domain (Serine/Threonine protein kinases(*S_TKc*)) (N-terminal ~30 kDa) and a series of *LCDs* (low complex domains) (Fig. 5F). So, this 55 kDa peptide belonged to the C-terminal of SNRK.

SNRK is related to the cell death and the junction of two domains contained an Asp residue (substrate of Caspases), both implying that SNRK might be degraded by Caspases [11]. Next, we cultured NRCMs with *Z-vad* (global inhibitor of Caspases) for 48 h, and discovered that the 55 kDa peptide is significantly downregulated (Fig. 5G). We further utilized SNRK as a bait to identify which Caspase (Caspase 3/6/7) functions here, and found that only cleaved-Caspase 3 is involved, which could pull the SNRK down (Fig. 5H). Finally, we decreased the Caspase 3 in NRCMs via *si-Caspase 3*, and the level of 55 kDa peptide reduced with time; however, the entire-SNRK increased (Fig. 5I). Taken together, these results demonstrated SNRK could be degraded by activated Caspase 3 into two peptides, and the 55 kDa peptide interact with NOVA1 directly.

Moreover, we transfected NRCMs with *pCMV-SNRK* or *pCMV-55kDa peptide* for 48 h followed *Z-vad* treatment to explore whether only 55 kDa peptide affects the formation of circ-SNRK. qRT-PCR showed that circ-SNRK only was impacted by the 55 kDa peptide (Fig. 5J). We also found that an NLS (nuclear localization sequence) locates at the C-terminal of 55 kDa peptide. Furthermore, immunofluorescence of anti-His showed that C-terminal peptide locates in both cytoplasm and nucleus, while the N-terminal peptide only residues in cytoplasm, suggesting that only 55 kDa peptide can enter the nucleus (Fig. 5K). Combined the fact that RNA splicing happens in nucleus, this finding provided a rationale for above phenomenon. To investigate how SNRK mediates the influences of NOVA1 on circ-SNRK formation, we up- or downregulated 55 kDa peptide in actinomycin D treated NRCMs and found that no obvious changes in protein NOVA1 is observed, suggesting that 55 kDa peptide has no effect on NOVA1 stability (Fig. 5L). We then utilized RIP of NOVA1 in NRCMs transfected with *pCMV-55kDa peptide* or *si-SNRK*; qRT-PCR showed that the pulled-down introns flanking circ-SNRK decreased in 55 kDa peptide overexpressed NRCMs, inferring that the interaction of NOVA1 with 55 kDa peptide could inhibit the binding of NOVA1 to introns (Fig. 5M). In conclusion, these data suggested that SNRK affects the formation of circ-SNRK by its 55 kDa peptide via NOVA1.

### Hypoxia increases the transcription of SNRK via *NF-κB* pathway

**Fig. 6:**
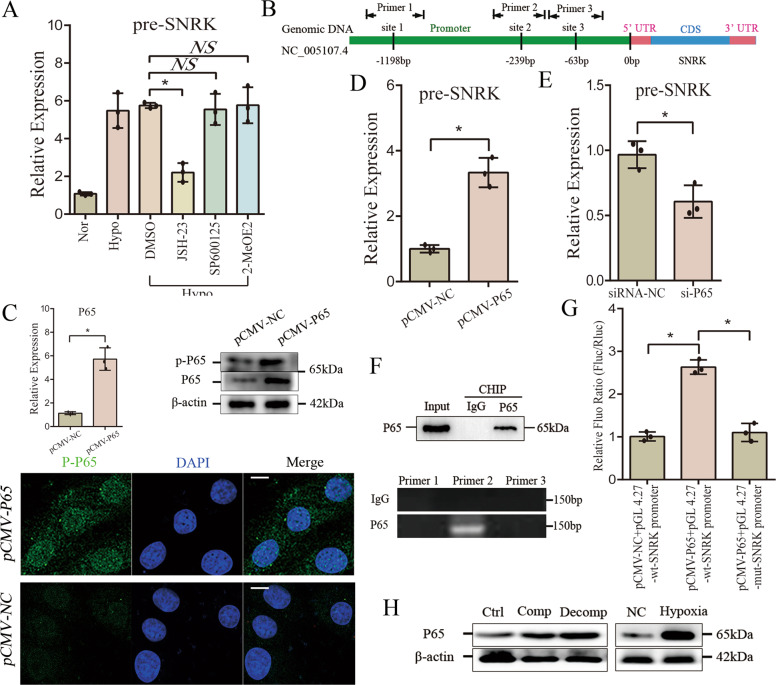
Hypoxia promotes the transcription of SNRK by the *NF-κB* pathway. **A** Bar graph showing the pre-SNRK in NRCMs treated with JSH-23, SP600125 or 2-MeOE2. **B** Representative schematic of the structure of SNRK promoter, and the 3 predicted binding sites of P65. **C** Upper: qRT-PCR (Left) and immunoblot analysis (Right) of P65 or p-P65 in NRCMs transfected with *pCMV-P65*; Lower: Immunofluorescence of P-P65 in NRCMs transfected with *pCMV-P65*. Scale bar = 5 um. **D** qRT-PCR of pre-SNRK in NRCMs transfected with *pCMV-P65*. **E** Bar graphs showing the pre-SNRK in NRCMs with *si-P65*. **F** CHIP assay of P65. (Upper) Immunoblot of P65 pulled down by anti-P65; (Lower) Agarose gel electrophoresis showing the sequences pulled down by P65. **G** Bar graph showing relative fluorescence intensity in NRCMs transfected with *pCMV-P65* and *pGL 4.27-wt-SNRK promoter* or *pGL 4.27-mut-SNRK promoter*. **H** Immunoblot analysis of P65 in hypoxia-treated NRCMs and HF heart. **A**, **C**–**E**, **G** All data were expressed as mean ± SD, *n* = 3, two-tailed *t*-test, *p < 0.05, NS not significant.

Our results suggested that hypoxia induces the changes of pre-SNRK, linear SNRK and circ-SNRK in CMs. To clarify the underlying mechanism, we analyzed the hypoxia-related transcription factors (TFs), including *HIF* (hypoxia-inducible factor), *NF-κB* (Nuclear factor NF-kappa-B) or *AP-1* (Activator protein-1) [22, 23]. To address which TF works here, three specific inhibitors: *JSH-23* (*NF-κB*), *SP600125* (JNK) and *2-MeOE2* (HIF-1α) were utilized, and results showed that the level of pre-SNRK significantly decreases in hypoxia-treated CMs only under the *JSH-23* treatment (Fig. 6A). Furthermore, bioinformatic analysis (*Jaspar* (http://jaspar.genereg.net/)) suggested three possible binding sites of *NF-κB* in the promoter of SNRK (Fig. 6B). Thus, we hypothesized that hypoxia might affect SNRK transcription through the *NF-κB* pathway.

Then, *pCMV-P65* (most abundant element in the *NF-κB* complex) was used to upregulate the level of P65. Immunofluorescence of p (phosphorylated) -P65 by confocal microscopy demonstrated an increase of nuclear P65 in *pCMV-P65* transfected NRCMs (Fig. 6C). The consequential significant increase of pre-SNRK in NRCMs confirmed the hypothesis that *NF-kB* complex could activate the transcription of SNRK (Fig. 6D); the reduction of pre-SNRK in *si-P65* transfected NRCMs further supported this notion (Fig. 6E).

Next, we designed three primers targeting the predicted sites of P65 in the promoter (Fig. 6B). CHIP (chromatin immunoprecipitation) assay of P65 was used to investigate their interaction. Results showed that sequences from the second site can be detected (Fig. 6F). We further cloned the *wt* or *mut* promoter of SNRK into *pGL 4.27 vector*. The fluorescence intensity in CMs co-transfected with *pCMV-P65* and *pGL 4.27-wt-SNRK promoter* was significantly higher than that in control groups (Fig. 6G). Finally, we found the increase of P65 in hypoxia-treated CMs or heart post-MI, demonstrating that pre-SNRK overexpression was associated with P65 in heart (Fig. 6H). Above all, we discovered that hypoxia could activate the transcription of SNRK via *NF-κB* pathway.

### Circ-SNRK improves cardiac function in MI-induced rats

**Fig. 7:**
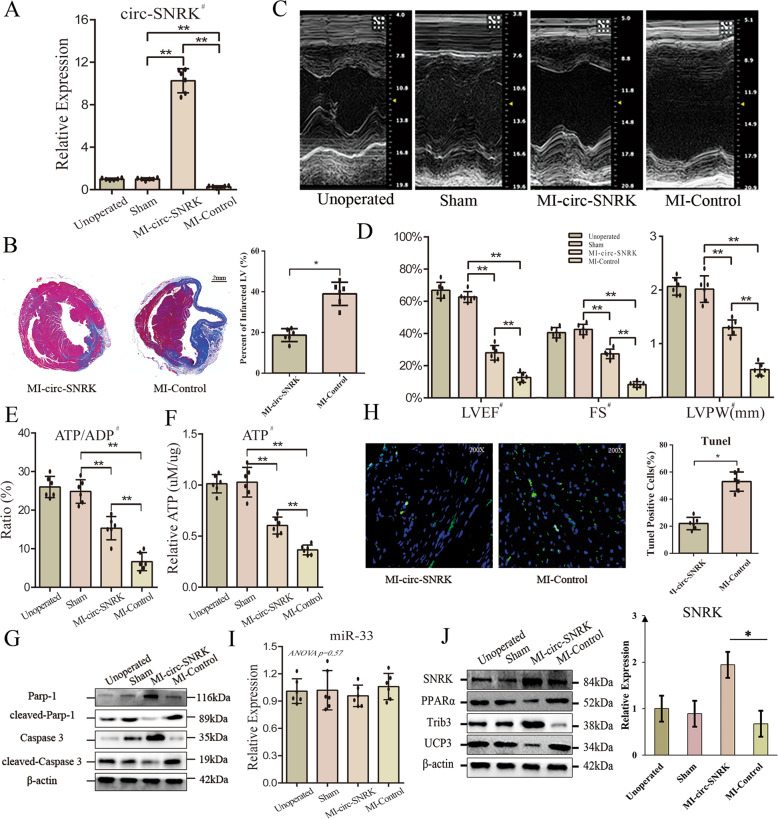
Circ-SNRK can improve cardiac function post-MI in rats. **A** qRT-PCR analysis of circ-SNRK in Unoperated, Sham, MI-circ-SNRK and MI-Control groups. **B** (Left) Representative images of hearts sections by Trichrome staining; **(**Right) Bar graph showing the infarcted LV in MI-circ-SNRK or MI-Control groups. Data were expressed as mean ± SD, *n* = *6*, two-tailed *t*-test, *p < 0.05. **C** Representative images of results of echocardiography from 4 groups. **D** Bar graphs of indexes (LVEF, LVPW and FS) in 4 groups. The ATP production (**F**) and ATP/ADP ratio (**E**) in 4 groups. **G** Immunoblot analysis of Caspase 3 and Parp-1 in Unoperated, Sham, MI-circ-SNRK and MI-Control groups. **H** (Left) Representative results of Tunel assay for heart samples; (Right) Bar graph showing the Tunel positive CMs per 100 cells in MI-circ-SNRK or MI-Control groups. Data were expressed as mean ± SD, *n* = *6*, two-tailed *t*-test, *p < 0.05 (**I**) qRT-PCR analysis of miR-33 in 4 groups. **J** (Left) Immunoblot of SNRK and related proteins in 4 groups; (Right) Quantification of protein SNRK with ImageJ. Data were expressed as mean ± SD, *n* = 3, two-tailed *t*-test, *p < 0.05. **A**, **D**–**F** All data were expressed as mean ± SD, *n* = 6, ^#^*one-way ANOVA p* < 0.05 of Sham, MI-circ-SNRK and MI-Control groups, **p < 0.01.

We next investigated whether circ-SNRK could improve the cardiac function post-MI in vivo. We established a HF rat model with overexpressed circ-SNRK (*MI-circ-SNRK group*) or not (*MI-Control group*) via viral vectors (AAV9). qRT-PCR demonstrated significant upregulation of circ-SNRK in MI-circ-SNRK group (Fig. 7A). Trichrome staining indicated the infarct size of the heart in MI-circ-SNRK group reduce remarkably (Fig. 7B). Furthermore, echocardiographic indexes (LVEF, FS and LVPW) were all higher in MI-circ-SNRK group, above suggesting a better cardiac function in circ-SNRK overexpressed rats (Fig. 7C, D). The increased ATP and ATP/ADP ratio in MI-circ-SNRK group verified its beneficial role in energy metabolism (Fig. 7E, F). In addition, immunoblot of Caspase 3/Parp-1 and Tunel assay confirmed that circ-SNRK could ameliorate the cell death in vivo (Fig. 7G, H). Finally, we investigated whether circ-SNRK in vivo exerted its functions through SNRK. Our data showed the expression of SNRK and related proteins (Trib3, PPARα, UCP3) are altered as expected. Meanwhile, no change in miR-33 was observed among different groups, consistent with our in vitro results (Fig. 7I, J). These results suggested that circ-SNRK can protect the heart from energy exhaustion via miR-33 - SNRK axis and in turn improves the cardiac function post-MI.

## Discussion

The development of post-MI heart failure contained two main stages: compensatory stage, marked by hypertrophy of left ventricle and a slight decline in EF; and decompensatory stage, characterized by ventricle dilatation and impaired contractility. Compensatory stage, an intermediate process, is characterized by unique physical and pathological traits. This stage ignored by previous studies was considered as an independent phase in our research, which was beneficial for us to find the ideal HF related circRNA. Here, we found a high percentage (98%) of differently expressed circRNAs in the Decomp group, co-existing in Ctrl and Comp group, had similar expression levels in these two groups. Given that hearts in these two groups had similar cardiac function, we hypothesized that these filtered circRNAs were closely associated with the regulation of cardiac function post-MI.

Circ-SNRK shared the miR-33 binding sites with linear SNRK. After excluding the degradation effect of miR-33, we questioned whether it blocks the translation of SNRK via binding to ORF (these shared sites are all on the ORF) directly. We co-transfected the NRCMs with *pCMV-SNRK*-*mut*-*3’ UTR* and miR-33; yet, no change was detected, suggesting that the binding of miR-33 to ORF does not affect translation (Supplementary Fig. S8I). A previous study showed that the translating ORF is protected by the ribosome complex from miRNAs, but the binding of miRNAs to 3’ UTR can inhibit the Cap-dependent translation via affecting the formation of translation loop [24]. Therefore, we concluded that miR-33 mainly affects the SNRK translation by binding to its 3’ UTR. As to the exogenous pre-circ-SNRK, the relatively low formation ratio (25%) of circ-SNRK made majority maintain the original form. Unlike the linear SNRK, the pre-circ-SNRK could sponge the miR-33 for the absence of protection from the ribosome complex. But, considering the higher degradation speed of pre-circ-SNRK, circ-SNRK likely exerted the major effects in CMs.

A previous study showed that SNRK protein reduces oxygen consumption and improves cardiac mitochondrial efficiency, which in turn decreases cell death [11]. Our data discovered that circ-SNRK could inhibit the cell apoptosis in vitro via UCP3 (key mediator of SNRK regulating energy metabolism) and reduce the heart infarct area in vivo, implying that circ-SNRK mainly impacts cell apoptosis via improving energy production (Fig. 7B/Supplementary Fig. S4/Supplementary Fig. S7C, D). Our work discovered that P65, the most enriched member of the *NF-κB* complex, binds to the promoter of SNRK and upregulates the pre-SNRK, the co-ancestor of linear SNRK and circ-SNRK. The hypoxia-activated P65 increased the circ-SNRK expression and in turn protected the CMs from cell death via SNRK, thus revealing another anti-apoptotic mechanism of *NF-κB* [22]. However, hypoxia accelerated the degradation of SNRK via Caspase 3, providing a balanced mechanism.

**Fig. 8:**
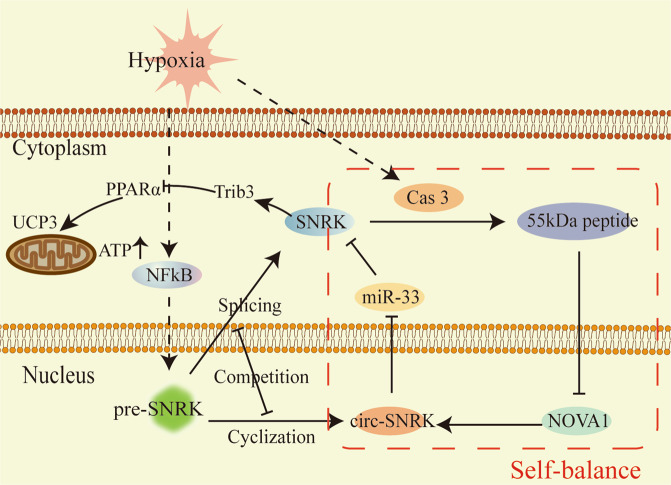
The graphical description of the negative feedback. A model depicting the negative feedback mechanism of SNRK and circ-SNRK in CMs.

The consequences of reduction in circ-SNRK were the downregulated SNRK via miR-33 and the upregulated SNRK for the formation competition of circ-SNRK with linear SNRK [18]. The latter effect can be ignored because the level of circ-SNRK is ~2% to linear SNRK. Meanwhile, the amplification effects of miR-33 to circ-SNRK (*7:1*) indicated that the former one dominates protein SNRK expression. Therefore, we found a negative feedback (Fig. 8): the translation of linear SNRK could be inhibited by miR-33; yet the reduction in protein SNRK weakens the translation inhibition of miR-33 by upregulating circ-SNRK via NOVA1. Thus, protein SNRK was limited at a proper level by this negative feedback. This mechanism also can explain why miR-33 increases the level of circ-SNRK, but not linear SNRK. The expression limitation of SNRK could be broken by overexpressed exogenous circ-SNRK. Our results in vivo demonstrated circ-SNRK overexpression is sufficient to improve the cardiac function post-MI in rats by breaking the feedback loop. Nevertheless, a recent study found that the level of miR-33 in fibroblast (another crucial member of the heart) significantly increase in pressure overloaded heart and further promote the cardiac fibrosis [25]. The upregulation of circ-SNRK in heart in vivo also could increase the circ-SNRK in fibroblasts and further inhibited the cardiac remodeling via sponging the miR-33 in fibroblasts, providing another explanation for the protective role of circ-SNRK in cardiac function post-MI.

In conclusion, the finding that NOVA1 could accelerate the circRNA formation associated alternative splicing factors with circRNA, extending our understanding of circRNA formation and directing the future research on circRNA. A feedback loop between protein SNRK and circ-SNRK limited the expression abundance of SNRK at a proper level; a bidirectional regulation of hypoxia in protein SNRK via P65 and Caspase 3, respectively, together suggesting that protein SNRK plays a vital role in the regulation of cardiac function post-MI. The introduction of exogenous circ-SNRK ameliorated the cell apoptosis in vitro and reduced CMs loss in vivo via SNRK, intensively emphasizing that circ-SNRK might be a new therapeutic target of HF in the future.

## Materials and methods

### Materials

Anti-PARP-1 (ab74290), Anti-Cleaved-PARP-1 (ab32064), Anti-Caspase 3 (ab13847), Anti-Cleaved-Caspase 3 (ab2302), Anti-β-actin, Anti-Caspase 6 (ab185645), Anti-Caspase 7 (ab255818), Anti-SNRK (ab96762), Anti-P62 (ab109012), Anti-LC3 (ab192890), Anti-Trib3 (ab73547), Anti-PPARα (ab215270), Anti-p-p65 (ab76302), Anti-p65 (ab16502), Anti-UCP3 (ab193470), Anti-IgG (ab133470), Anti-His (ab18184) are purchased from Abcam (Britain). TRIzol reagent was acquired from Invitrogen (USA); an SYBR RT-PCR Kit and DNA PCR kit were from Takara Bio Inc. (Japan); RNase R was from Epicenter (USA). RNA-Binding Protein Immunoprecipitation Kit was purchased from Millipore (USA). T7 RNA polymerase and RNase-free DNase I were bought from Promega (USA).

Primers, mimic, and siRNAs were designed and synthesized by Sangon Biotech (China). Rho123, Caspase 3 activity Kit, CCK-8 Kit and ATP Assay Kit were purchased from Beyotime (China). A Dual-luciferase reporter assay system was purchased from Promega (USA). *Z-vad, JSH-23, SP600125, 2-MeOE2* were purchased from Selleck (USA).

### Animal model and samples

A total of 49 male SD rats, 6 weeks old, weighing ~200 g, were obtained from Vital River Laboratories, China. All the animals were housed in an environment with temperature of 22 ± 1 °C, a relative humidity of 50 ± 1% and a light/dark cycle of 12/12 h. All animal studies (including the rats euthanasia procedure) were done in compliance with the regulations and guidelines of the Research Institute of Medicine of Shanghai Jiao Tong University institutional animal care and conducted according to the AAALAC and the IACUC guidelines.

All animals were anesthetized using 10% chloral hydrate, intubated with a 16-gauge trachea cannula and ventilated with an animal respirator. The left anterior descending coronary artery was then ligated with a 6–0 nylon silk suture after the heart was exposed. The MI model was considered successfully established when there was a change in color (pallor was observed on the anterior wall of the left coronary artery). Based on the *echocardiographic* measurements and general shapes of hearts, rats were classified into 3 groups (3 rats/group): control group (Ctrl, without surgery; LVEF > 55%, 1.6 mm < LVPW < 2.8 mm and 25% < FS < 46%), compensatory group (Comp, with surgery; LVEF > 55%, LVPW > 2.8 mm and 25% < FS < 46%) and decompensatory group (Decomp, with surgery; LVEF < 55%, LVPW < 1.6 mm and FS < 25%) [13, 14]. The hearts were collected postoperatively at 4 (Ctrl and Comp group) and 6 weeks (Decomp group) and preforming RNA sequencing. In addition, to examine whether circ-SNRK was associated with aging, hearts and other organs (brain, muscle, liver, spleen, lung, kidney, and testis) were collected from healthy rats at different age (hearts were collected from 1 day, and 1, 3, 4, 5 months-old rats; other organs were collected from 3 month-old rats).

Additional 40 SD male rats (6 weeks old) were randomly categorized into 4 groups (10 rats/group): Unoperated group that received no surgery; Sham group, which received sham surgery (no ligation was performed); and two experimental groups (MI-Control group with AAV9-NC and MI-circ-SNRK group with AAV9-circ-SNRK), which were treated with 5*10^11^ viral genomes (vg) containing SNRK (Obio Technology, Shanghai, China) or empty vector before LAD ligation. General features of rats are shown in Supplementary Table S3.

After 6 weeks, all the rats underwent echocardiographic evaluations by a blinded tester. The rats were then sacrificed by cervical dislocation. All samples were stored in the liquid nitrogen for further use.

### Cell culture and cell treatments

Primary NRCMs (neonatal rat cardiomyocytes) were isolated as previously described [26]. Briefly, 1- to 3-day-old SD rats were euthanized by decapitation, their hearts were collected, and the ventricles were minced and digested in 0.1% collagenase. Cell suspensions were then collected, centrifuged, resuspended in DMEM with 10% FBS, 100 U/ml penicillin and 100 μg/ml streptomycin, and plated for 1.5 h under standard culture conditions (humidified atmosphere containing 5%CO_2_/95% air at 37 °C), which allows fibroblast attachment to the culture plates. Consequently, cells in suspension (mostly NRCMs) were collected and cultured for an additional 24 h. Monoclonal antibodies against α-actin were used to identify cardiomyocytes. Cell purity was confirmed by both examining cellular morphology (beating cells) and immunostaining, revealing that the percent of NRCMs was over 90% [26]. These cells were tested negative for mycoplasma contamination by PCR analysis.

### Circular RNA high-throughput sequencing and computational analysis

RNAs were sent to the RIBOBIO (Guangzhou China) for high-throughput sequencing, which was performed on an Illumina HiSeq 3000 platform in PE150 sequencing mode. Quantile normalization and subsequent data processing were performed using the R software package and analyzed by RIBOBIO. The computational pipeline CIRC explorer was used to obtain back-spliced junction reads for circRNAs prediction; the expression abundance of circRNAs was measured based on back-spliced reads per million mapped reads (reference genome Rnor_6.0). The raw data had been uploaded to the NCBI (SRP340001).

### Annotation for circRNA/miRNA/mRNA interactions

The circRNA/miRNA/mRNA interactions were predicted with Arraystar’s home-made miRNA target prediction software based on miRanda (microrna.org/microrna/home.do) and TargetScan (targetscan.org/vert_71/) as previously described [27]. The match score was set to be higher than 150 and the minimum free energy less than −25 to improve the reliability of our prediction.

### Gene Ontology (GO) analysis and Kyoto Encyclopedia of Genes and Genomes (KEGG) pathway analysis

GO analysis (geneontology.org) was used to construct meaningful annotation of gene products in three domains, including biological process (BP), cellular components (CC) and molecular function (MF). KEGG (genome.jp/keg/) was performed to predict the molecular interactions and reaction networks associated with differentially regulated genes. The enrichment score (-log_10_ (P-value)) represented the significance of GO term enrichment or pathway enrichment among circRNA targets or genes which produced differentially expressed circRNA.

### Quantitative real-time polymerase chain reaction (qRT-PCR) analysis and reverse-transcription PCR (RT-PCR) analysis

Total RNAs isolated from tissues and cells were reverse transcribed into cDNA for qRT-PCR analysis (Takara Bio Inc, Japan), which was conducted in ABI QuantStudio™ 6 Flex Real-time PCR systems in accordance with the manufacturer’s instructions. Relative expression of 2^(-ΔΔCT)^ compared to the value of control was used to analyze the gene expression. Agarose gel electrophoresis was used to analyze the results of RT-PCR. The following primers were used for the experiment: circ-SNRK—5’-AGTTGACAGACTTTGGCTTC/AAAAGTGCTTGAAGTGGGTC-3’, pre-SNRK —5’-GCTTCAGCAACAAGTTTCAG/ACTTTGAAATAACCTGTCTGCC-3’, linear SNRK—5’-GCTTCAGCAACAAGTTTCAG/TCAGCGTCTCACTGTCGTTG-3’, NOVA1—5’-GAGAATTACTTCCATCTCAAC/GATGCTACATGATGAACTAAA-3’, DDX1—5’-TTCCCAGGTTGAGCCA/CAACTCTTGAACGGTAGG-3’, RED1-5’-CCCAATCTGTCCGTAGC/TCACAACTCTTTCCATCCC-3’.

### ATP assay

The intracellular ATP level of CMs and tissues was determined by an ATP Assay Kit according to the manufacturer’s instructions (Beyotime, China).

### ATP/ADP measurements

The ATP/ADP levels in cells and tissues were measured using bioluminescent detection (ADP/ATP Ratio Assay Kit, Abcam) according to the manufacturer’s instructions. The bioluminescent intensities were measured on a multi-mode microplate reader (Synergy H1 Hybrid, BioTek).

### Determination of the mitochondrial membrane potential (MMP)

Rho123 (Beyotime, China), which can bind specifically to mitochondria, has been used in numerous investigations to estimate MMP with some modifications [28]. The low MMP increases the fluorescence intensity of Rho123 in cells and vice versa. In this study, treated cells were harvested, washed, and then incubated with Rho123 (5 mg/ml) in PBS for 60 min in the dark at 37 °C. The fluorescence was measured by flow cytometry.

### Immunoblot

The cells were washed with PBS, lysed on ice in lysis buffer supplemented with protease inhibitors and phosphatase inhibitors and then centrifuged at 12,000 × *g* for 15 min at 4 °C. The resulting cell lysates were resolved on 7.5% or 12.5% SDS-PAGE gels and then transferred to PVDF membranes. These membranes were blocked in 5% non-fat dry milk in TBST with 0.1% Tween-20 for 1.5 h at room temperature before the appropriate primary antibodies were incubated with the membranes overnight at 4 °C. The membranes were subsequently washed and incubated with a horseradish peroxidase (HRP)-conjugated secondary antibody for 1 h at room temperature, after which the antibody complexes were visualized and quantified using a chemiluminescence western blotting detection system (Tanon, Shanghai, China). The protein expression levels of the target genes were quantified by relative densitometry and normalized to bands corresponding to β-actin, which were used as an internal control. The results were analyzed by Image J. The experiment was run in triplicate.

### MiRNA, plasmid transfection and RNA interference

To achieve the gain or loss of circ-SNRK, miR-33, SNRK, NOVA1 and 55 kDa peptide, NRCMs were transfected *pGV486-circ-SNRK* (2ug), *si-circ-SNRK* (50 nM), miR-33 mimics (50 nM), miR-33 inhibitors (100 nM), *Ad (Adenovirus)-SNRK* (mol = 5), *si-SNRK* (50 nM), *pCMV-NOVA1* (2ug), *si-NOVA1* (50 nM), and *pCMV-55kDa peptide* (2ug) (RiboBio, Guangzhou, China), respectively using Lipofectamine 3000 (Invitrogen, USA), according to the manufacturer’s instructions. Control cells were transfected with the appropriate negative controls. Adenovirus was used to increase the protein levels because the primary cell had low transfection efficiency with plasmids. We designed two siRNAs targeting different region of one gene to avoid the off-target effects. The sequences of the oligonucleotides were as follows: si-circ-SNRK: 5’-TTGCTGTTTCTCCTACTGCAGG-3’, si-SNRK: 5’-TCAGCGTCTCACTGTCGTTG-3’, si-NOVA1: 5’-GATGCTACATGATGAACTAAA-3’, Si-DDX1: 5’-GAAGGTAGCCAAGGTGTAGGA-3’, Si-RED1: 5’-AAGCACGGTACCTGCTCACAA-3’.

### Northern blotting

Northern blotting was performed according to the manufacturer’s instructions (DIG Northern Starter Kit, Roche). Digoxigenin (Dig)-labeled antisense probes targeting the junction of circ-SNRK were designed by Sangon Biotech (China). In brief, 5 ug of total RNA was resolved on denaturing urea polyacrylamide gel, transferred to nylon membrane (Roche) and UV-crosslinked using standard manufacturer’s protocol. Membrane was then hybridized with specific Dig-labeled RNA probes.

### RNA immunoprecipitation (RIP)

RIP assay was performed using a Magna RIP RNA-Binding Protein Immunoprecipitation Kit (Millipore, USA) according to the manufacturer’s instructions. Antibodies for RIP assays against AGO2, NOVA1 and IgG were purchased from Abcam (USA).

### Dual-luciferase reporter assay

To construct a luciferase reporter vector, we synthesized fragments of the SNRK 3’ UTR, circ-SNRK, promoter of SNRK and their mutant sequences, which were then inserted into a pmirGLO. For the reporter assay, we co-transfected NRCMs that were seeded in 24-well plates with either luciferase reporter construct and miR-33 mimic, or with negative control. At 48 h after transfection, the cells were lysed, and luciferase activity levels were measured using a dual-luciferase reporter assay system (Promega, USA). Firefly luciferase activity levels were normalized to Renilla luciferase activity levels. The experiment was repeated three times.

### RNA pull-down assay

Biotin-labeled predicted RNAs or NC RNAs were transcribed with Biotin RNA Labeling Mix (47) and T7 RNA polymerase (Promega. USA), treated with RNase-free DNase I (Promega, USA), and purified using RNeasy Mini Kit (Qiagen, German). Biotin-labeled RNAs were mixed and incubated with NRCMs extracts. Streptavidin-conjugated magnetic beads (Invitrogen, USA) were added to each binding reaction and further incubated. Beads were then thoroughly washed, and the retrieved proteins were detected by Western blot.

### Quantification and statistical analysis

All data acquisition and analysis were performed by investigators blinded to experimental group. For biochemical analyses, a minimum of four samples per genotype were used for each analysis, while in vivo analysis included at least six rats per genotype. These sample sizes are sufficient to determine whether there is a biologically meaningful difference between different genotypes, given the known rat-to-rat variation in energy metabolism and cell apoptosis assessments in previous studies. As for in vitro studies, a sufficient large number of cells were analyzed to ensure the description of biologically meaningful differences, also following the methods from studies cited throughout the paper. Moreover, results obtained in cells were reliably reproduced in at least three independent experiments. All experimental data were expressed as mean ± SD unless otherwise mentioned. Normality of the variables was tested by means of the Shapiro–Wilk test. The data from the analysis met the assumptions of the tests and the variance was similar between the experimental groups (if not, the data was not included in the following analysis). Unpaired two-tailed Student´s t test was used when comparing two experimental groups, while three experimental groups were analyzed using *one-way ANOVA* followed by Tukey’s post hoc test. The Prism program version 7.0 (GraphPad Software Inc.) was used for calculations and P values lower than 0.05 were considered significant. Data were recorded and analyzed with SPSS 22.0 (IBM, Chicago, IL, USA), and a *P* value of less than .05 was considered statistically significant.

## Supplementary information

Fig.S1

Fig.S2

Fig.S3

Fig.S4

Fig.S5

Fig.S6

Fig.S7

Fig.S8

Fig.S9

Table S1

Table S2

Table S3

## Data Availability

The datasets used and/or analyzed during the current study are available from the corresponding author on reasonable request.
