# Supplementary figures and images for: Negative feedback of SNRK to circ-SNRK regulates cardiac function post-myocardial infarction
Source: Cell Death Differ. 2021 Oct 7;29(4):709–21. doi: 10.1038/s41418-021-00885-x (PMC8989981; doi:10.1038/s41418-021-00885-x)

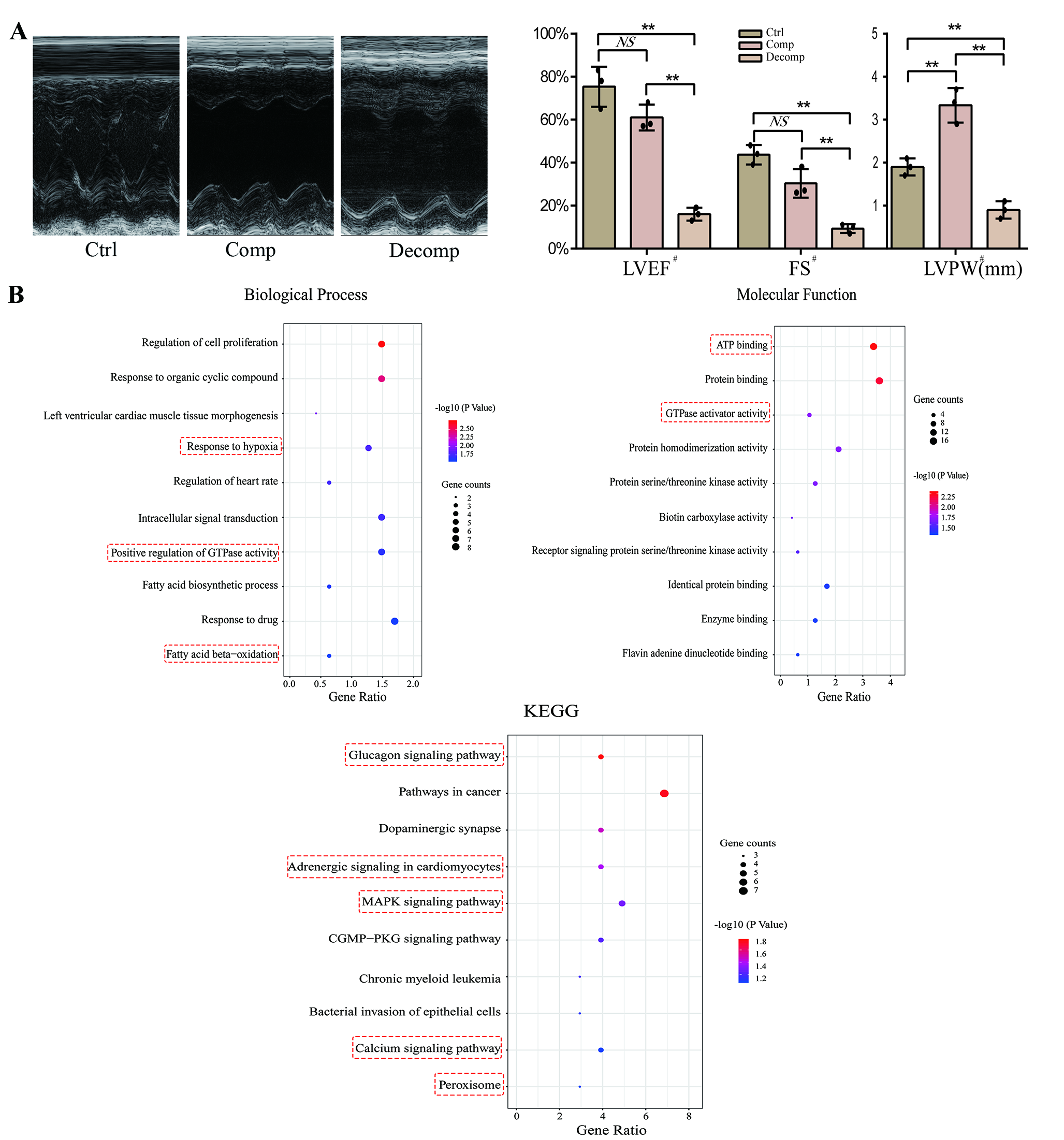

Supplement: Supplementary file 1 — Fig.S1 [file 41418_2021_885_MOESM1_ESM.tif]

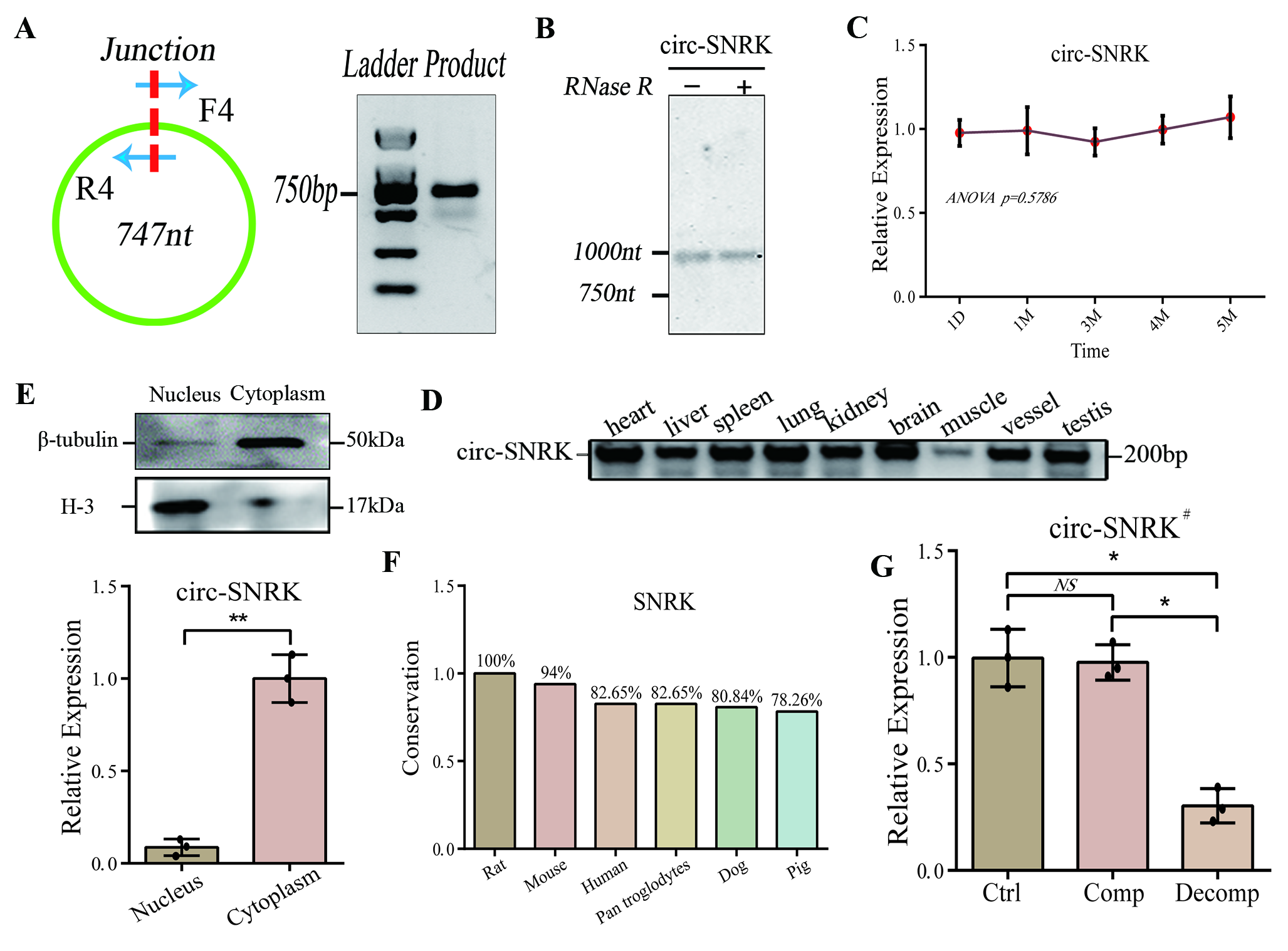

Supplement: Supplementary file 2 — Fig.S2 [file 41418_2021_885_MOESM2_ESM.tif]

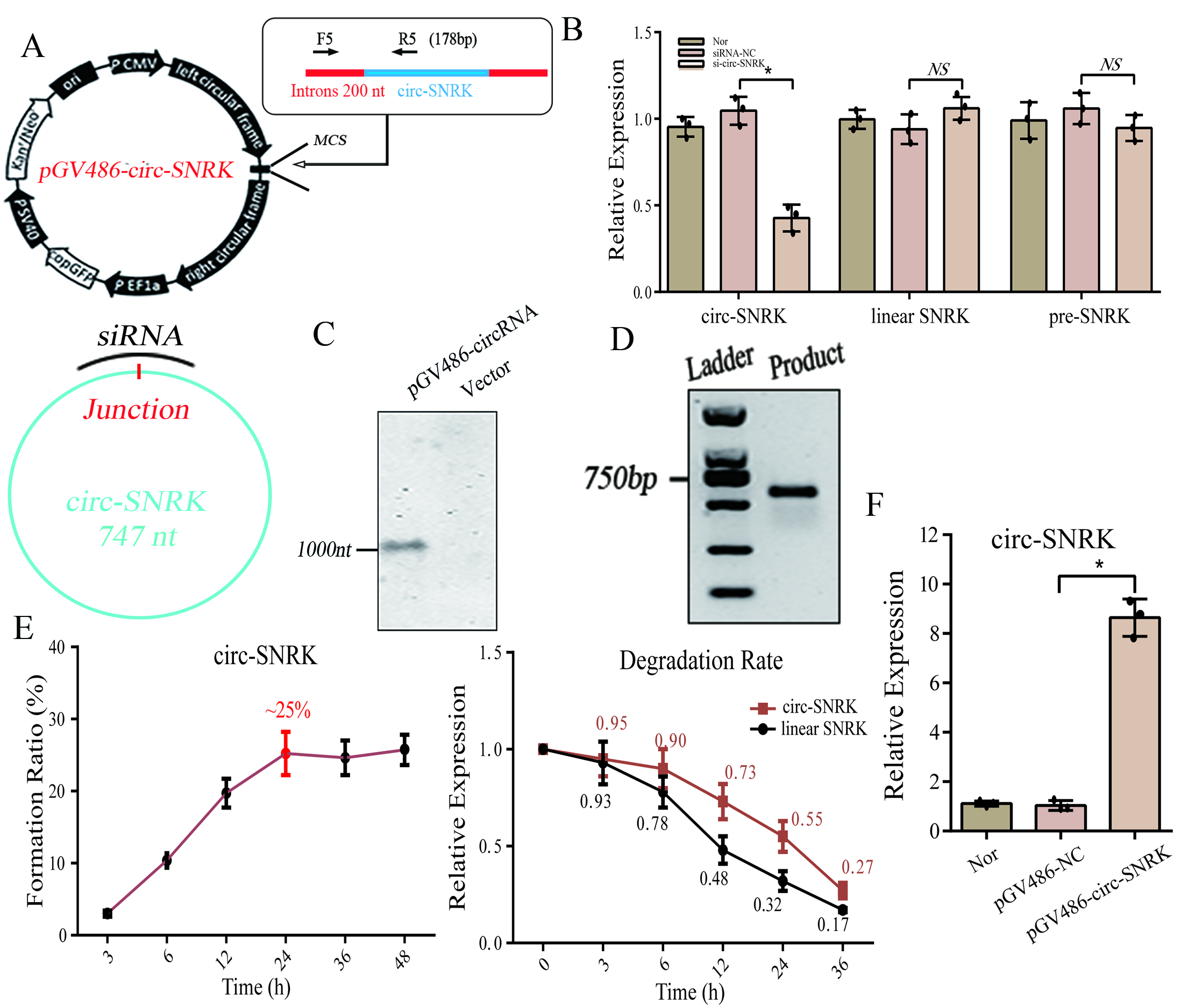

Supplement: Supplementary file 3 — Fig.S3 [file 41418_2021_885_MOESM3_ESM.tif]

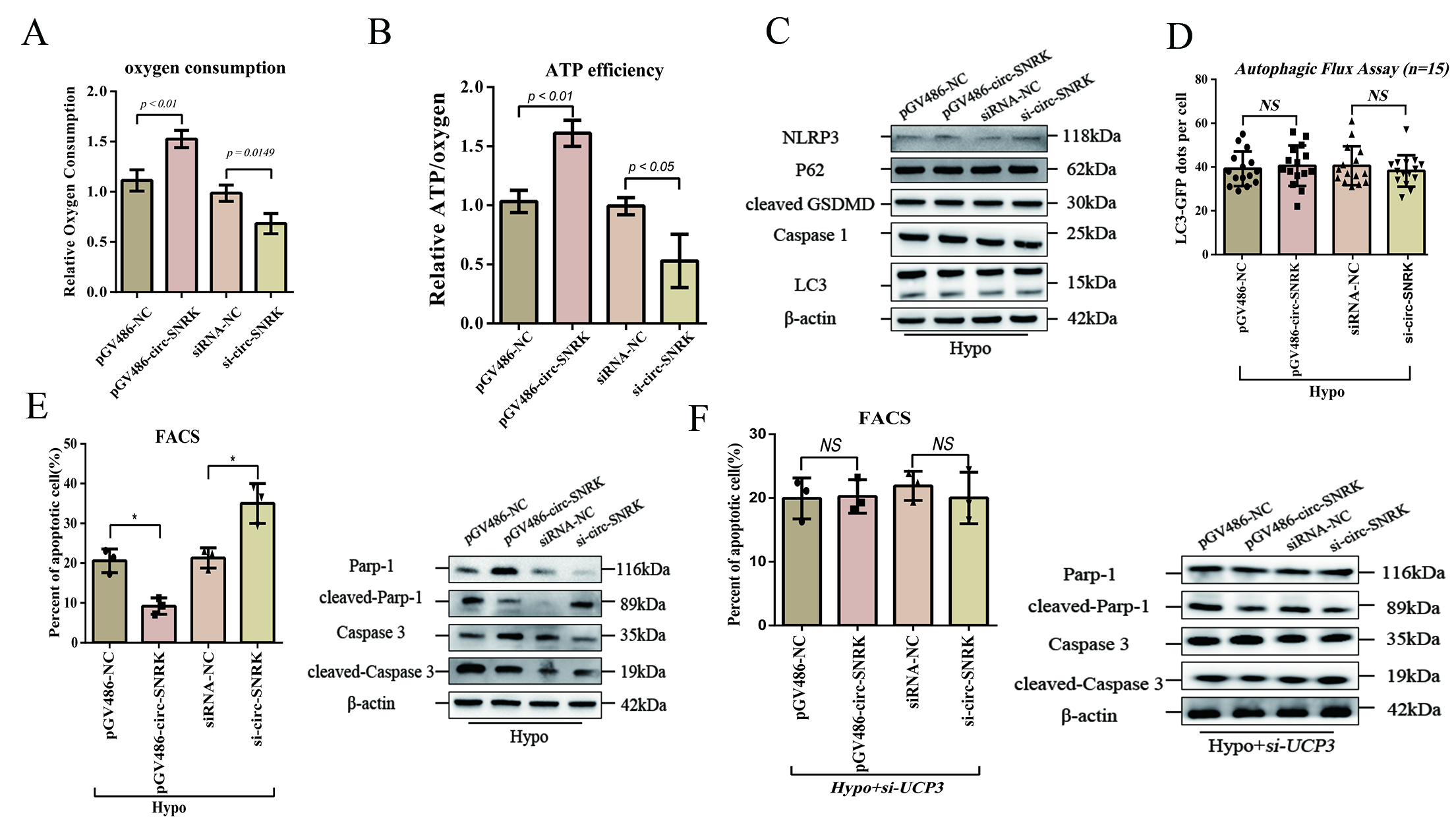

Supplement: Supplementary file 4 — Fig.S4 [file 41418_2021_885_MOESM4_ESM.tif]

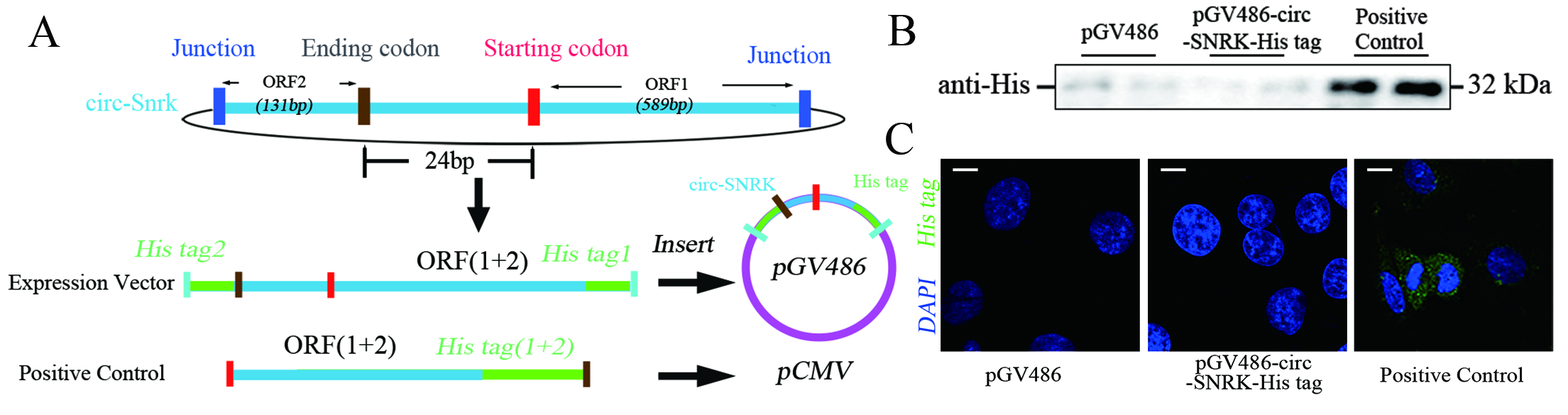

Supplement: Supplementary file 5 — Fig.S5 [file 41418_2021_885_MOESM5_ESM.tif]

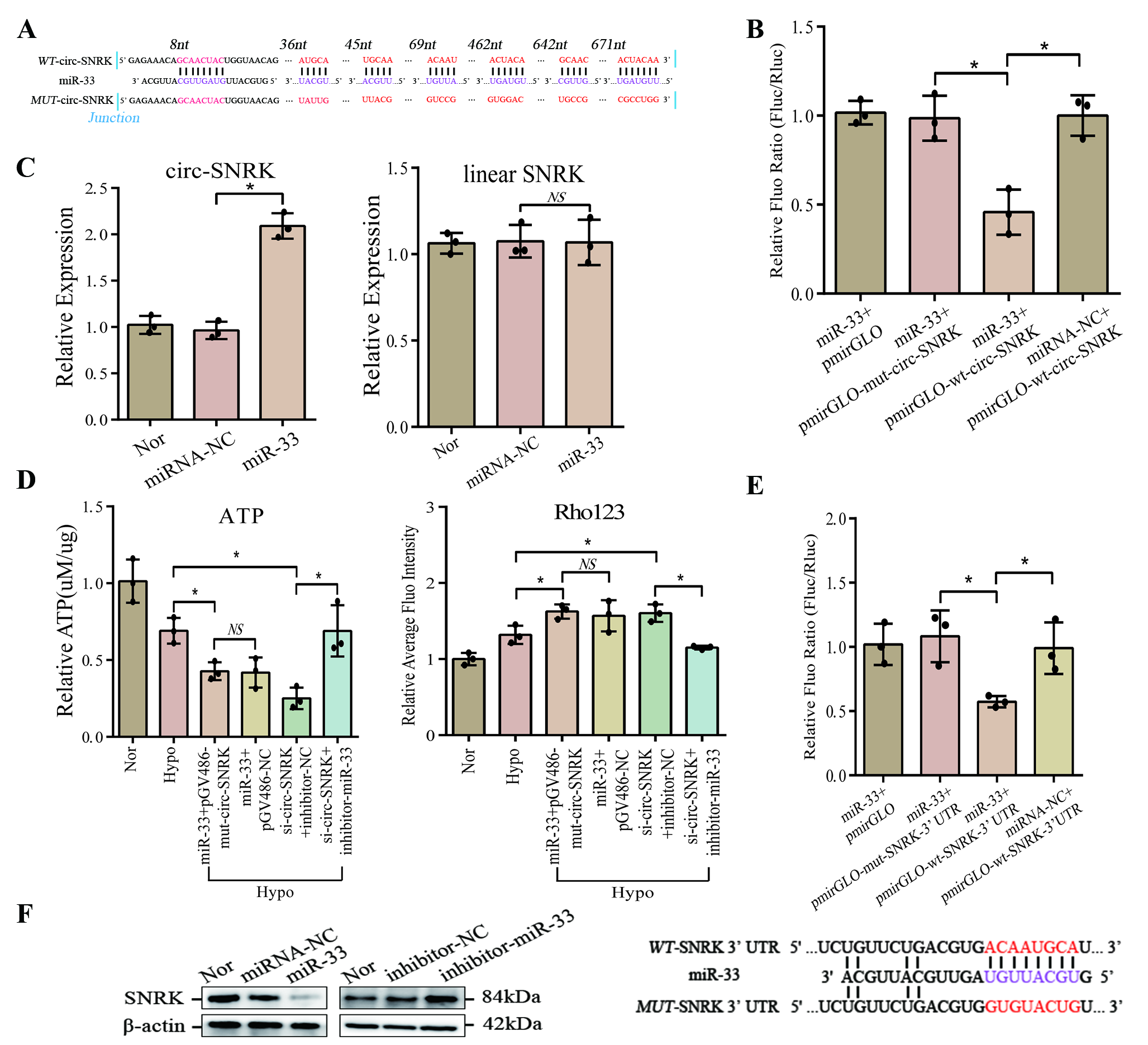

Supplement: Supplementary file 6 — Fig.S6 [file 41418_2021_885_MOESM6_ESM.tif]

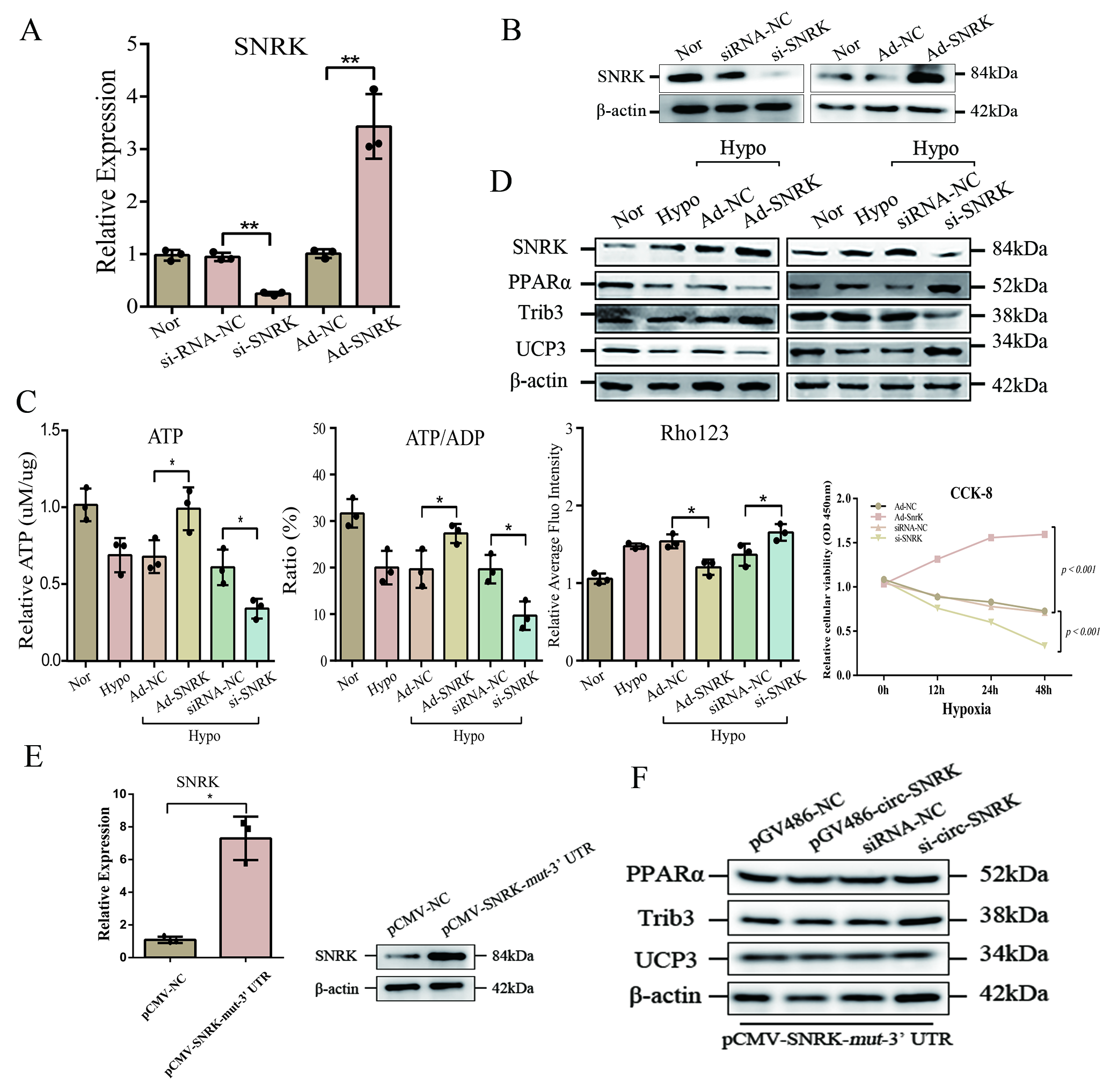

Supplement: Supplementary file 7 — Fig.S7 [file 41418_2021_885_MOESM7_ESM.tif]

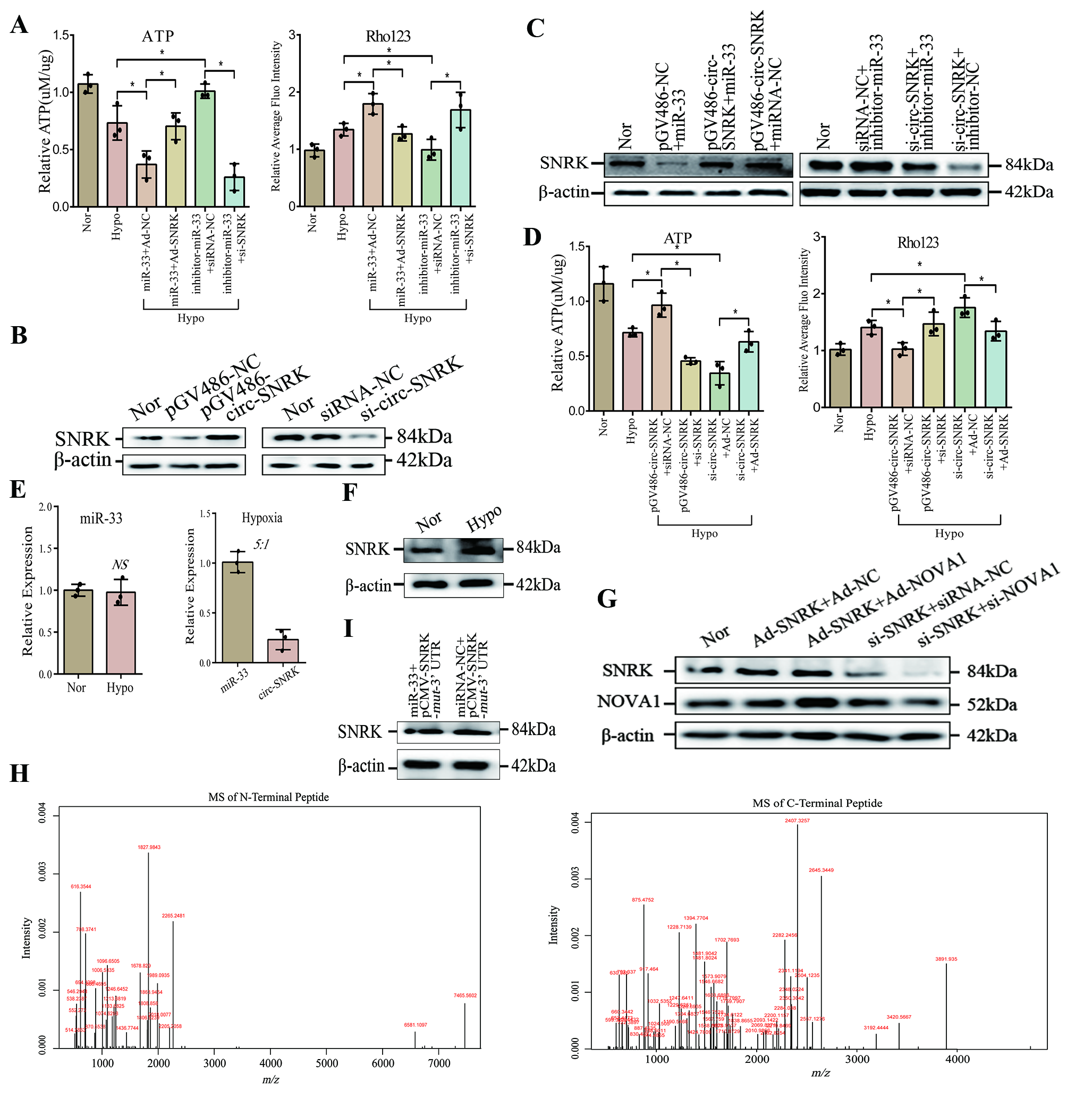

Supplement: Supplementary file 8 — Fig.S8 [file 41418_2021_885_MOESM8_ESM.tif]

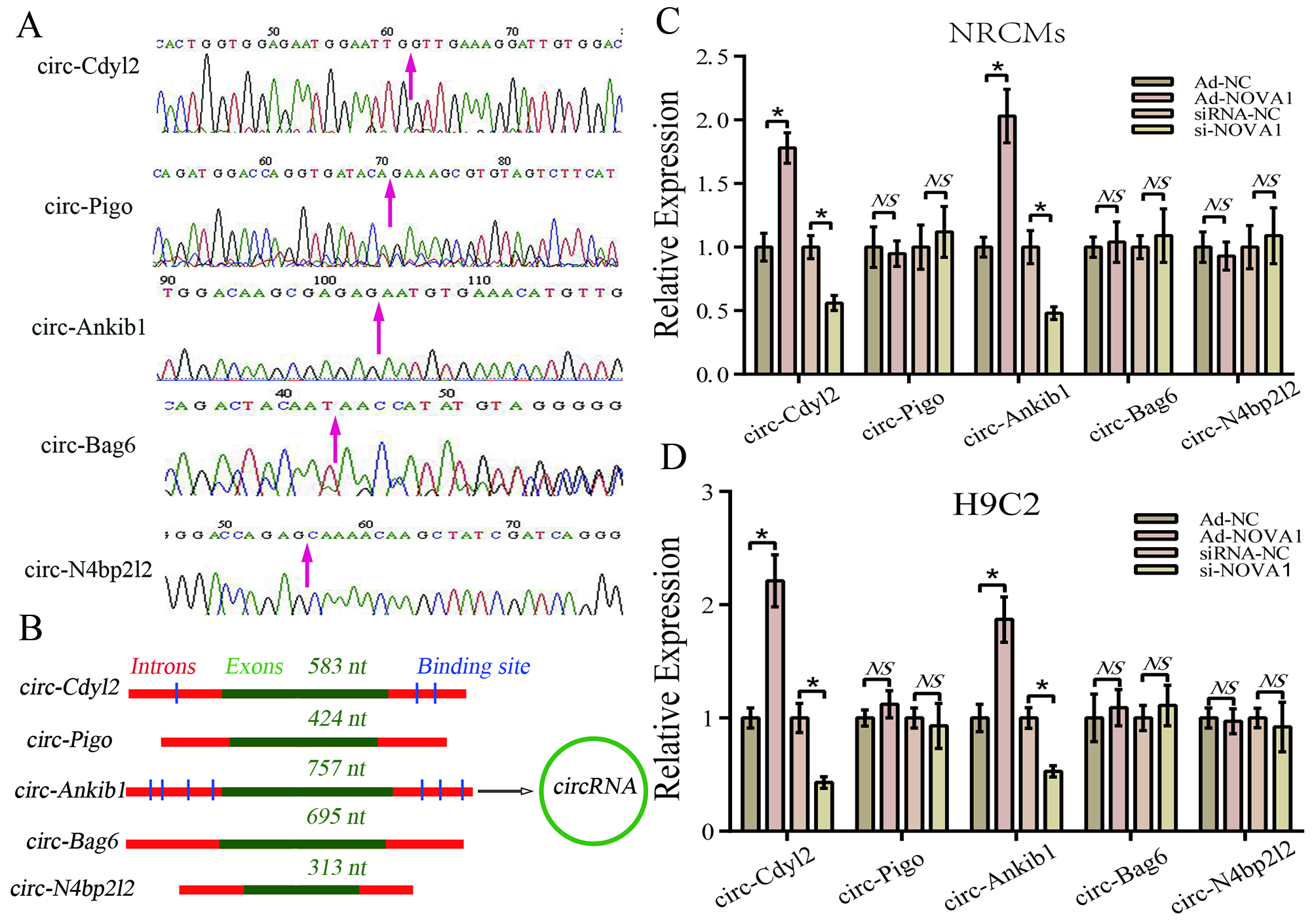

Supplement: Supplementary file 9 — Fig.S9 [file 41418_2021_885_MOESM9_ESM.tif]
