# Supplementary material for: Negative feedback of SNRK to circ-SNRK regulates cardiac function post-myocardial infarction
Source: Cell Death Differ. 2021 Oct 7;29(4):709–21. doi: 10.1038/s41418-021-00885-x (PMC8989981; doi:10.1038/s41418-021-00885-x)
Supplement: Supplementary file 12 — Table S3 [file 41418_2021_885_MOESM12_ESM.docx]

| **Table S3. General Features of Rats** | | | | | | | | | | |
| --- | --- | --- | --- | --- | --- | --- | --- | --- | --- | --- |
|  | 0 week | | | | |  | 6 weeks | | | |
|  | Un-op | Sham | | MI-C | MI-S |  | Un-op | Sham | MI-C | MI-S |
| Number | 10 | 10 | | 10 | 10 |  | 10 | 10 | 7 | 8 |
| Age (weeks) | 6 | 6 | | 6 | 6 |  | 12 | 12 | 12 | 12 |
| Body weight (BW, g) | 209±15 | 198±21 | | 201±17 | 193±23 |  | 378±25 | 389±31 | 289±21* | 323±29* |
| Mortality | - | - | | - | - |  | 0 | 0 | 30% | 20% |
| Heart W/BW (mg/g) | - | | - | - | - |  | 3.21±0.12 | 3.12±0.17 | 4.12±0.31* | 3.71±0.28* |
| *Note: Un-op = unoperated; MI-C = MI-Control; MI-S = MI-circ-SNRK; * p < 0.05 VS Un-op* | | | | | | | | | | |
